# COmpressioN following endovenous TreatmenT of Incompetent varicose veins by sclerotherapy (CONFETTI)

**DOI:** 10.1016/j.jvsv.2023.101729

**Published:** 2023-12-09

**Authors:** Amjad Belramman, Roshan Bootun, Tristan R.A. Lane, Alun H. Davies

**Affiliations:** a Section of Vascular Surgery, Department of Surgery and Cancer, Imperial College London, London, United Kingdom; b Faculty of Medicine, Omar Al-Mukhtar University, Derna, Libya; c Vascular Surgery Specialty Training Registrar in the East of England Deanery, London, United Kingdom; d Cambridge Vascular Unit, Cambridge University Hospitals NHS Foundation Trust, London, United Kingdom; e Department of Vascular Surgery, Imperial College Healthcare NHS Trust, London, United Kingdom

**Keywords:** Compression, Pain, Postoperative, Treatment outcomes, Varicose veins

## Abstract

**Objective:**

The evidence for post-foam sclerotherapy compression stockings for varicose veins is limited. Thus, we examined the effects of post-procedural compression stockings on varicose vein patients undergoing foam sclerotherapy.

**Methods:**

The CONFETTI study was a prospective, single-center, randomized controlled trial. Patients with foam sclerotherapy-suitable varicose veins were randomly assigned to the compression group (CG) or the no compression stockings group (NCG) for 7 days. The primary outcome was post-procedural pain measured on a 100-mm visual analog scale for 10 days. Secondary outcomes included clinical severity, generic and disease-specific quality of life scores, return to normal activities and/or work, occlusion rates, degree of ecchymosis, CG compliance, and complications. Patients were reviewed at 2 weeks and 6 months.

**Results:**

A total of 139 patients were consented to and randomly assigned. The intention-to-treat analysis included 15 patients who did not receive the allocated intervention. Both groups had similar baseline characteristics. Of the patients, 63.3% and 55.4% returned for follow-up at 2 weeks and 6 months, respectively. Most of the veins treated were tributaries. The CG experienced significantly lower pain scores than the NCG, with median scores of 7 mm and 19 mm, respectively (Mann-Whitney *U*-test; *P* = .001). At 2 weeks, no differences were observed in ecchymosis or the time to return to normal activities or work. Both groups showed improvements in clinical severity and quality of life, and occlusion rates were comparable. The NCG experienced one deep venous thrombosis and superficial thrombophlebitis, whereas the CG experienced two superficial thrombophlebitis.

**Conclusions:**

The CONFETTI study suggests that short-term post-procedural compression stockings are beneficial for reducing post-procedure pain.

**Article Highlights**

• **Type of Research:** A single-center, prospective, randomized controlled trial
• **Key Findings:** A total of 139 patients were randomly assigned to either a compression group or a no compression group, with 69 and 70 patients, respectively. Results showed that the compression group experienced significantly lower pain scores than the no compression group, with median scores of 7 mm and 19 mm, respectively (Mann-Whitney *U*-test; *P* = .001).
• **Take Home Message:** Short-term post-procedural compression stockings are beneficial for reducing post-procedure pain, but their long-term benefits remain unclear.

Varicose veins (VVs) of the lower extremities are a prevalent problem, affecting up to one-third of adults and significantly lowering their quality of life (QoL). Patients with VVs may experience pain, achiness, swelling, heaviness, itching, and even a leg ulcer.^1^ Invasive surgery such as ligation and stripping is no longer the preferred treatment option for VVs. Instead, endovenous ablation techniques (EATs) like endovenous laser therapy (EVLT) and radiofrequency ablation (RFA) have demonstrated clinical effectiveness, cost-effectiveness, and fewer complications.^2^ Ultrasound-guided foam sclerotherapy (UGFS) is a chemical EAT that has been in use for years, but long-term results show it is less effective than other EATs.^3^ The National Institute for Health and Care Excellence (NICE) recommends thermal techniques (TTs), including EVLT and RFA, and foam sclerotherapy, as the preferred treatment options, respectively.^4^ UGFS can be implemented in both truncal and tributary veins, and it is especially useful for tortuous recurrent veins and anatomical configurations that make endovenous cannulation or ablation difficult.^5^

The postoperative management of VVs remains unclear. As demonstrated by the Vascular Society of Great Britain and Ireland (VSGBI) surveys, most surgeons routinely use bandages postoperatively, with 46% employing elastic bandages,^6^ and the mean duration for compression being 7 days (range, 2 days to 3 months).^7^ These surveys highlighted the variability of United Kingdom practice and urged more research.

Numerous researchers have investigated the practice of using compression after foam sclerotherapy. Hamel-Desnos et al^8^ found no difference in efficacy, side effects, satisfaction scores, symptoms, or QoL between the treatment groups (31 received Class II stockings and 26 did not). According to Campos Gomes et al,^9^ applying elastic compression for 3 weeks did not diminish the rate of post-intervention reflux in either group (those receiving vs those not receiving elastic compression), but it did decrease the saphenous vein diameter. Other studies could not determine which compression regimens are superior.^10^^,^^11^

A systematic review demonstrated insufficient evidence to support the use of post-procedural compression stockings and was unable to determine the most effective compression or duration required.^12^ Tan et al similarly found that compression following sclerotherapy potentially has benefits in the short-term; although, again, there was insufficient evidence regarding the duration, compression strength, and type of compression.^13^

The American Venous Forum (AVF), Society for Vascular Surgery (SVS), American College of Phlebology (ACP), Society for Vascular Medicine (SVM), and International Union of Phlebology (UIP) clinical practice guidelines all recommend compression therapy immediately after sclerotherapy treatment of superficial veins to improve outcomes (Grade: 2; Level of Evidence: C).^14^ The SVS and AVF also suggested using post-procedural compression for 1 week to prevent hematoma formation, pain, and swelling.^15^ The NICE guidelines (2013) recommended using post-procedural compression for no more than 7 days after VV intervention,^4^ although NICE also proposed more research on clinical efficacy, cost effectiveness, and optimal compression duration.^4^

Therefore, the COmpressioN Following Endovenous TreatmenT of Incompetent Varicose Veins by Sclerotherapy (CONFETTI) study was devised to investigate the effects of post-procedural compression stockings following endovenous ablation using foam sclerotherapy.

## Methods

### Study design and population

The CONFETTI study was a prospective, single-center, randomized controlled trial (RCT) that was conducted at the Local Anesthetic Varicose Veins Unit located at Charing Cross Hospital, United Kingdom (Imperial College Healthcare NHS Trust). The eligibility criteria for participation included adult patients with VVs who were suitable and had consented for UGFS treatment, whereas the exclusion criteria are outlined in the Supplementary Table (online only). A flow diagram illustrating the study design and participant enrollment process is provided in the Supplementary Fig (online only).

### Randomization

Patients were required to provide signed informed consent for the research study prior to undergoing the UGFS procedure. Subsequently, they were randomly assigned to one of two groups: a compression group (CG) or a no compression group (NCG). Patients were assigned using an online randomization program called Sealed Envelope Ltd.^16^ Although participant blinding was not feasible in this study, the clinicians performing the procedure and the assessors conducting follow-up assessments and duplex ultrasound (DUS) were blinded to the treatment allocation.

### Procedures

Before undergoing the procedure, all patients in the study underwent an assessment of their clinical severity scores and were required to complete validated questionnaires to assess their QoL. The UGFS procedure was conducted by experienced vascular surgeons using the sclerosant sodium tetradecyl sulphate (STS). The clinicians involved had discretion over the volume and concentration of the injected foam and were blinded to the treatment group allocation.

The procedure involved identifying the target vein using DUS and instilling sclerosant foam prepared using a 4:1 mixture of STS (STD Pharmaceutical Products) and air, following the Tessari method.^17^ No concomitant intervention was performed during the procedure.

Following the injection of sclerosant foam, the deep venous system and veins treated were assessed using DUS. All patients were provided with thigh-length elastic bandages (Bastos Viegas) to wear for 24 hours after the procedure. Patients who were randomly assigned to the CG were additionally provided with thigh-length class II compression stockings (18-24 mmHg) (Credalast, Credenhill Limited). The size of the compression stockings was estimated by measuring the leg circumference at four points (upper and lower leg, ankle, and knee) by a vascular specialist nurse.

Patients in the CG were instructed to wear the compression stockings for 24 hours a day, 7 days a week, except when showering, and to record their compliance in a diary provided. No compression stockings were provided to patients in the NCG. Prior to discharge, all patients were given a diary to record their postoperative pain for 10 days using a 100-mm visual analog scale (VAS), and to report their compliance with wearing compression stockings and the resumption of normal activities/work. Patients were encouraged to mobilize and resume normal activities as soon as possible. No routine post-procedural analgesia was prescribed. The number of cannulations, level of cannulation, veins treated, and volume of sclerosant foam used were documented.

### Follow-up

Two weeks after the procedure, patients underwent clinical assessment to identify any potential complications, severity scores, or ecchymosis. Patients also completed QoL questionnaires, and their diaries were collected. Patients in the CG were asked about their compliance with wearing compression stockings. At 6 months, clinical severity scores were reassessed, and patients completed QoL questionnaires again. The occlusion rate was determined using DUS at the 6-month follow-up.

### Outcomes

The aim of this study was to examine the impact of post-procedural compression stockings on patients who underwent endovenous ablation using UGFS. The primary outcome measure was the level of post-procedural pain, which was evaluated using a 100-mm VAS daily for a period of 10 days. Secondary outcome measures included changes in clinical severity scores, QoL scores, time taken to return to normal activities and/or work, occlusion rates at 6 months, degree of ecchymosis,^18^ compliance with wearing compression stockings in the CG, and any complications that may arise.

Using the venous clinical severity score (VCSS), the venous disability score (VDS), and^19^ the clinical severity of venous disease was evaluated. Additionally, the Clinical, Etiologic, Anatomic, and Pathophysiologic (CEAP) classification was documented. QoL was measured using both generic and disease-specific questionnaires, including the Aberdeen varicose vein questionnaire (AVVQ) and the chronic venous insufficiency quality of life questionnaire (CIVIQ-14),^20^, ^21^, ^22^ the EuroQol 5 Domain 3 Level (EQ-5D-3 L) and EuroQol VAS.^23^^,^^24^

### Power calculations

The design of this RCT resembles that of the COMETA trial,^25^ which was conducted within our unit. The initial power calculation was based on estimated number from similar investigations^8^^,^^26^^,^^27^ focused on compression therapy following foam sclerotherapy. This calculation was based on the enrolment of 350 patients, with the objective of detecting a 10-mm difference on the VAS with a standard deviation of 20 mm. The parameters used included 90% power, 5% significance, and an estimated dropout rate of 50%.

However, due to recruitment rates falling below anticipated levels, a revision of the power calculation was necessary. Consequently, the study proceeded with a reduced sample size of 128 patients, ensuring 80% power and 5% significance (64 participants per group), following ethical committee amendment approval.

### Statistical analysis

All collected data was entered into a database, and subsequent analyses were performed on an intention-to-treat basis using SPSS Statistics version 27.0.1 (IBM Corp) and Stata Statistical Software version 17.0 (StataCorp). The normality of continuous data distribution was assessed using visual inspection, the Kolmogorov-Smirnov, and the Shapiro-Wilk tests. Appropriate statistical tests, such as the Student *t*-test or Mann-Whitney *U* test, were then employed based on the data’s distribution. Paired data was analyzed using related samples of Friedman’s two-way analysis of variance or one-way repeated measures analysis of variance. Categorical data were analyzed using the Pearson χ^2^ test or the Fisher exact test. The statistical significance level was set at *P* < .05.

### Ethics and registration

The North East - Newcastle and North Tyneside 1 Research Ethics Committee (Reference: 15/NE/0314) approved the study, which was conducted in accordance with the Helsinki Declaration. The study was sponsored by Imperial College London (Reference number: 15HH2894, IRAS project ID: 187,992), which registered at ClinicalTrials.gov (NCT02655406) and the ISRCTN registry (ISRCTN17719156).

### Funding

The study was supported by a research fellowship funded by the Union Internationale de Phlebologies/Chemische Fabrik Kreussler & Co GmbH.

## Results

Recruitment for the study was conducted from January 15, 2016, to January 31, 2019, during which 139 patients consented and were randomly allocated into two groups: NCG (n = 70) and CG (n = 69). The mean age of the patients was 57.7 ± 14 years, with 53% of them being women. Of the 139 patients, 15 did not receive their allocated intervention due to reasons such as a preference for compression hosiery or current treatment (a short stretch bandage), cancellation of the procedure, or withdrawal from the study. These patients were included in the analysis on an intention-to-treat basis.

**Fig 1:**
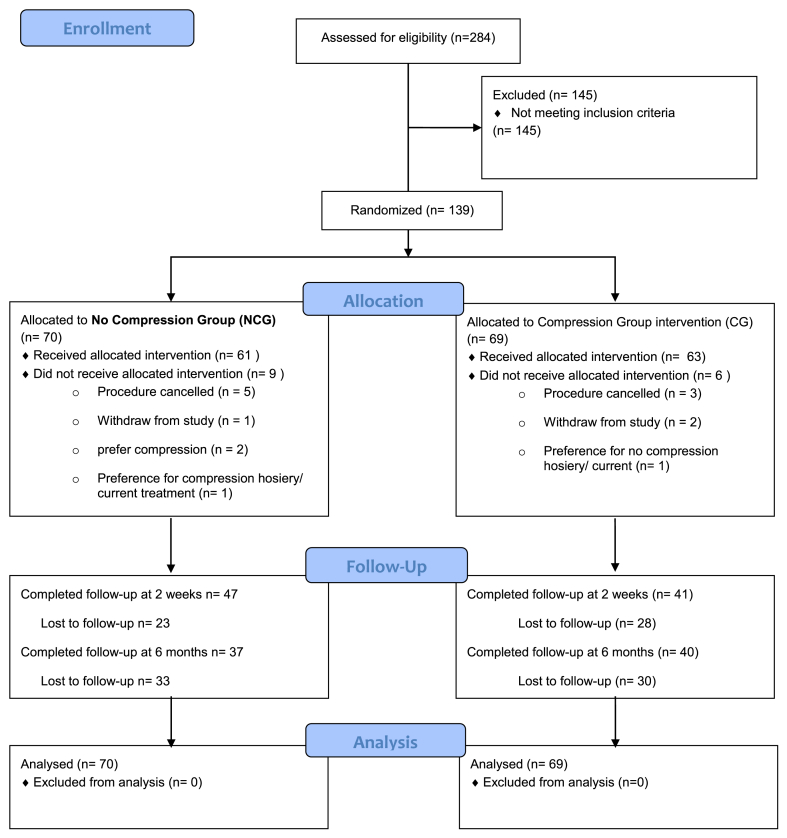
Study Consolidated Standards of Reporting Trials (*CONSORT*) diagram. *CG*, Compression group; *NCG*, no compression group.

Both groups had comparable baseline characteristics (Table I). The median clinical scores using the VCSS in the CG were 4 (interquartile range [IQR], 3-5), whereas in the NCG, it was 4 (IQR, 3-6) (*P* = .951). Baseline generic and disease specific QoL did not show any statistically significant differences (Table II). Follow-up assessments were conducted at 2 weeks and 6 months, with 63.3% and 55.4% of the patients returning, respectively (see the Consolidated Standards of Reporting Trials (CONSORT) diagram in Fig 1).

**Table I:** Patients’ baseline characteristics

Characteristics	Overall (N = 139)	NCG (n = 70)	CG (n = 69)	*P*-value
Age, years	57.7 (±14)	57.0 (±14)	58 (±14)	.897^a^
Sex				
Female	101 (72.7)	54 (53.5)	47 (46.5)	.233^b^
Height, meters	1.67 (±0.92)	1.67 (±0.95)	1.68 (±0.90)	.949^a^
Weight, kg	77.7 (±20.3)	79.8 (±.20)	75.7 (±20)	.602^a^
BMI, kg/m^2^	27.1 (±7.2)	28.5 (±7.5)	26.9 (±6.8)	.855^a^
BMI >30 kg.m^2^	37 (35.9)	18 (35.3)	19 (36.5)	.895^b^
Smoker	23 (18.1)	9 (14.5)	14 (21.5)	.304^b^
Hypertension	22 (17.1)	12 (19)	10 (15.2)	.556^b^
Previous treatment of VVs	82 (59)	43 (61.4)	39 (56.5)	.549^b^
Clinical CEAP class^d^				
C2	56 (42.7)	28 (43.1)	28 (42.4)	.868^b^
C3	39 (29.8)	21 (32.3)	18 (27.3)	
C4	32 (24.4)	14 (21.5)	18 (27.3)	
C5	4 (3.1)	2 (3.1)	2 (3)	
Clinical severity scoring				
VCSS	4 (3-6)	4 (3-6)	4 (3-5)	.951^c^
VDS	1 (1-2)	1 (1-2)	1 (1-2)	.414^c^
Generic QoL				
EQ-VAS	80 (70-90)	80 (70-92)	80 (70-90)	.388^c^
EQ-5D	.73 (.66-.76)	.76 (.65-.76)	.71 (.67-.76)	.468^c^
Disease-specific QoL				
AVVQ	17.95 (11-25.3)	17.7 (9.9-26.6)	18.2 (11.2-24.1)	.968^c^
CIVIQ-14	25 (12.5-44.6)	23.2 (10.7-40.6)	28.5 (14.2-53.5)	.220^c^

*AVVQ*, Aberdeen Varicose Vein Questionnaire; *BMI*, body mass index; *CEAP*, Clinical Etiology Anatomy Pathology; *CG*, compression group; *CIVIQ-14*, Chronic Venous Insufficiency Quality of Life Questionnaire; *EQ-5D*, Euroqol 5 Domain 3 Level; *EQ-VAS*, EuroQoL’s Visual Analogue Scale; *NCG*, no compression group; *QoL*, quality of life; *VCSS*, Venous Clinical Severity Score; *VDS*, Venous Disability Score.

Data are presented as number (%), mean (± standard deviation), or median (interquartile range).

a Student *t*-test.

b χ^2^ test.

c Mann-Whitney *U* test.

d Not all randomly assigned patients received the intervention.

**Table II:** Characteristics of the veins treated

Characteristics	Overall (N = 139)	NCG (n = 70)	CG (n = 69)	*P*-value
Leg treated	.237^a^			
Right	61 (45.5)	33 (50.8)	28 (40.6)	
Veins treated^b^				
Tributaries	98 (70.5)	46 (65.7)	52 (75.3)	.864^a^
ATV	6 (4.3)	3 (4.3)	3 (4.3)	
SSV	5 (3.59)	3 (4.3)	2 (2.89)	
GSV	13 (9.3)	5 (7.14)	8 (11.59)	
Cannulation level^c^				
Above knee	27 (24.5)	13 (25)	14 (24.5)	.291^a^
Below knee	60 (54.5)	25 (48)	35 (60)	
Above & below knee	23 (20.9)	14 (27)	9 (15.5)	
Volume of foam used, mL	8 (5-10)	10 (5-10)	5 (5-10)	.022^b^
Number of cannulations	1 (1-2)	1 (1-2)	1 (1-2)	.427^b^

*ATV*, Anterior thigh vein; *CG*, compression group; *GSV*, great saphenous vein; *NCG*, no compression group; *SSV*, small saphenous vein.

Data are presented as number (%), mean (± standard deviation), or median (interquartile range).

a χ^2^ test.

b Mann-Whitney *U* test.

c Missing data.

Table II provides a summary of the treated veins, revealing that the NCG received a significantly higher median volume of sclerosant foam compared to the CG (10; IQR, 5-10 vs 5; IQR, 5-10; *P* = .022, Mann-Whitney test). Both groups had a similar proportion of patients who had undergone previous VV treatment, with tributaries being the most frequently treated vein type (varicosities).

### Primary outcome (pain score)

**Fig 2:**
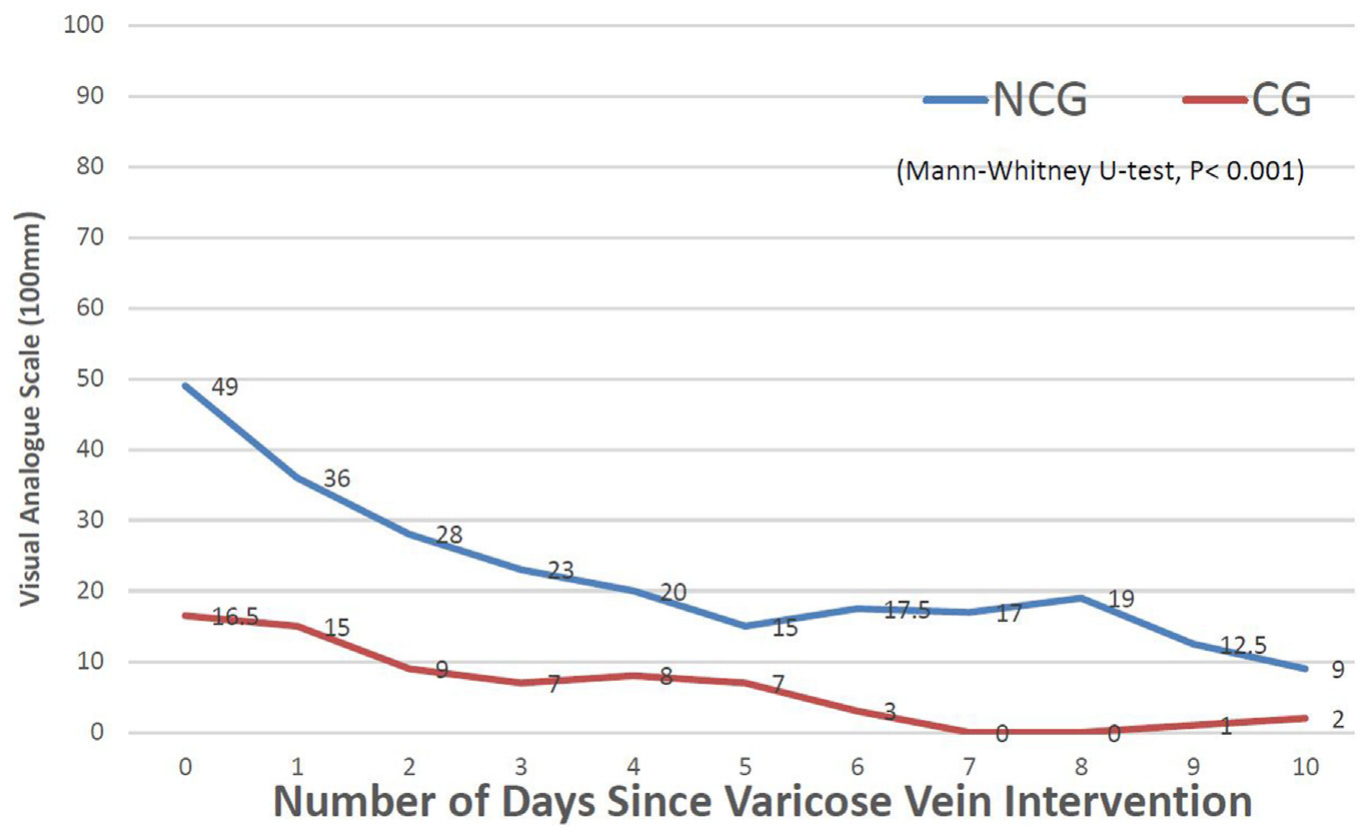
The median postoperative pain score in the first 10 days postoperatively in the no compression group (*NCG*) and compression group (*CG*) measured with the visual analogue scale (VAS).

During the first 10 days, the VAS pain scores were low in both groups. However, the CG patients experienced significantly lower pain scores compared with the NCG patients, as evidenced by a median of 7 mm (IQR, 1-9 mm) in the CG vs 19 mm (IQR, 15-28 mm) in the NCG (Mann-Whitney *U* test; *P* = .001). These results are visually represented in Fig 2.

### Secondary outcomes

Table III summarizes the secondary outcomes.

**Table III:** Summaries of secondary outcomes

Secondary outcomes	Overall	NCG (n = 70)	CG (n = 69)	*P*-value
Clinical severity scoring				
VCSS				
Baseline (n = 133)^c^	4 (3-6)	4 (3-6)	4 (3-5)	.951^b^
2 weeks (n = 88)^c^	3 (2-5)	3 (2-4)	4 (2-6)	.103^b^
6 months (n = 75)^c^	3 (2-6)	3 (2-5)	4 (2.7-6)	.185^b^
VDS				
Baseline (n = 132)^c^	1 (1-2)	1 (1-2)	1 (1-2)	.414^b^
2 weeks (n = 87)^c^	1 (0-1)	1 (0-1)	1 (0-1)	.330^b^
6 months (n = 74)^c^	1 (0-1)	0 (0-1)	1 (0-1)	.373^b^
Generic QoL				
EQ-VAS				
Baseline (n = 123)^c^	80 (70-90)	80 (70-92)	80 (70-90)	.388^b^
2 weeks (n = 82)^c^	85 (70-94)	83.5 (70-93)	89 (74.7-95)	.373^b^
6 months (n = 77)^c^	80 (63-90)	85 (70-95)	79.5 (60-90)	.036^b^
EQ-5D				
Baseline (n = 133)^c^	.73 (.66-.76)	.76 (.65-.76)	.71 (.67-.76)	.468^b^
2 weeks (n = 84)^c^	.76 (.66-.80)	.76 (.68-.81)	.76 (.65-0.83)	.971^b^
6 months (n = 75)^c^	.76 (.65-.96)	.76 (.69-1)	.69 (.65-76)	.013^b^
Disease-specific QoL				
AVVQ				
Baseline (n = 130)^c^	17.95 (11-25.3)	17.7 (9.9-26.6)	18.2 (11.2-24.1)	.968^b^
2 weeks (n = 86)^c^	17.6 (10-25.4)	13.5 (8.1-24)	19.5 (13.1-26.4)	.103^b^
6 months (n = 74)^c^	15.8 (6.9-23.6)	11.47 (6.1-23.8)	17 (9.3-23)	.290^b^
CIVIQ-14				
Baseline (n = 127)^c^	25 (12.5-44.6)	23.2 (10.7-40.6)	28.5 (14.2-53.5)	.220^b^
2 weeks (n = 86)^c^	17.5 (8.9-39.2)	16 (8.9-33.9)	19.6 (12.5-44.6)	.374^b^
6 months (n = 69)^c^	19.6 (8-39.2)	16.9 (3.57-30.8)	19.6 (12.5-44.6)	.233^b^
Compliance with wearing compression	_	_	8 (5-10)	
Return to normal activities	2 (0-3)	2 (1-3)	1 (0-3)	.479^b^
Return to work	2 (1-5)	2 (1-4)	2 (1-6)	.331^b^
Complete occlusion rates at 6 months				
56 (40.2%)				
Complete occluded	32	13 (40.6)	19 (59.4)	.249^a^
Partially occluded	24	13 (54.2)	11 (45.8)	
Ecchymosis				
<25%	67 (94.4)	22 (97.4)	13 (90.6)	.266^a^
≥25%	4 (5.6)	1 (2.6)	3 (9.4)	

*AVVQ*, Aberdeen Varicose Vein Questionnaire; *CIVIQ-14*, Chronic Venous Insufficiency Quality of Life Questionnaire; *CG*, compression group; *EQ-5D*, EuroQoL 5 Domain 3 Level; *EQ-VAS*, EuroQoL Visual Analogue Scale; *NCG*, no compression group; *QoL*, quality of life; *VCSS*, Venous Clinical Severity Score.

Data are presented as number (%) or median (interquartile range).

a χ^2^ test.

b Mann-Whitney *U* test.

c Number of questionnaires completed by patients.

#### Clinical disease severity–VCSS and VDS

During the follow-up period of 2 weeks to 6 months, both VCSS and VDS exhibited a statistically significant improvement in both groups (VCSS, χ^2^(2) = 7.635; *P* = .022; (VDS, χ^2^(2) = 49.214; *P* = .000, Friedman). However, there was no statistically significant difference observed in either the VCSS or VDS scores between the two groups.

#### Quality of life

Regarding the generic QoL using EQ-5D and EQ-VAS, both groups demonstrated significant improvements in their scores from baseline to 6 months. The Friedman test results showed no significant differences between the groups at 2 weeks for either EQ-VAS (χ^2^(2) = 4.899a; *P* = .086) or EQ-5D (χ^2^(2) = 2.150; *P* = .341). However, at 6 months, the NCG had better EQ-5d and EQ-VAS scores than the CG.

Regarding the disease-specific QoL, the AVVQ score improved significantly in both groups after 2 weeks, and these improvements were maintained for up to 6 months (χ^2^(2) = 8.393; *P* = .015, Friedman). Post hoc analysis revealed that the AVVQ scores decreased significantly from baseline (median, 17.9) to 6 months (median, 15.8) (*P* = .035), but not at 2 weeks (median, 17.6) (*P* = 1.000). Similarly, the CIVIQ-14 scores decreased in both groups from baseline, but there was no significant difference between the two groups (χ^2^(2) = 4.939; *P* = .085, Friedman test).

#### Wearing compression stockings

Among the patients in the CG, 45% reported adhering to wearing compression stockings for a median duration of 8 days, with an IQR of 5 to 10 days. Interestingly, there was no significant difference found between patients who were compliant with the compression stockings and those who were not.

#### Ecchymosis

At the 2-week follow-up, the extent of ecchymosis was evaluated using a 5-point grading scale ranging from 0% to 100%. The grading categories were divided into two groups: those covering 25% or less and those covering more than 25%. Analysis revealed that 94.4% of patients in both groups had ecchymosis that covered 25% or less of the treatment area. Although the data indicated that patients in the CG had less ecchymosis compared with the NCG, this difference was not statistically significant (90.6% vs 97.4%; 13 in the CG and 22 in the NCG: χ^2^; *P* = .266).

#### Return to normal activities and work

Overall, the median time for patients to return to normal activities or work was 2 days. Specifically, patients in the NCG returned to normal activities after a median duration of 2 days (IQR, 1-3 days), whereas those in the CG returned to normal activities after a median duration of 1 day (IQR, 0-3 days). However, the difference was not statistically significant (*P* = .479). Similarly, the median duration for patients to return to work was 2 days for both the NCG (IQR, 1-4 days) and the CG (IQR, 1-6 days), and this difference was also not statistically significant (*P* = .331).

#### Occlusion rates

A 6-month DUS was performed on 56 patients, which accounted for 40.2% of the total sample. Analysis of the results indicated that the rates of complete vein occlusion were comparable between the two groups. Specifically, 13 patients (40.6%) in the NCG and 19 patients (59.4%) in the CG achieved complete vein occlusion (χ^2^; *P* = .249, with Yates’ correction of *P* = .375).

#### Complications

The study revealed that the NCG encountered a single case of deep vein thrombosis (DVT) and one case of superficial thrombophlebitis (SVT), whereas the CG had two cases of SVT. One patient in the NCG developed DVT 2 weeks after undergoing treatment for medial thigh and calf varicosities and was prescribed direct oral anticoagulants (DOACs) after a calf DVT was confirmed. Another patient in the NCG who received sclerotherapy injections for left distal calf varicosity developed SVT 6 months after treatment. In contrast, the CG had a 67-year-old man with a history of recurrent SVT who experienced another episode of SVT and was prescribed rivaroxaban for 10 days. The remaining case of SVT occurred in a 72-year-old woman. Additionally, minor complications such as phlebitic reactions, skin pigmentation, and an erythematous, hard area at the injection site were reported, which are known to occur with foam sclerotherapy.

## Discussion

The CONFETTI study, which is one of the largest RCTs investigating the impact of post-procedural compression following UGFS, has revealed that wearing compression stockings provides a significant benefit in terms of pain reduction in the short-term compared with the NCG. However, uncertainties still exist regarding any potential advantages in terms of QoL, time to return to normal activities or work, occlusion rates, and complications.

The analysis reveals a significant reduction in post-procedure pain scores among patients in the CG. This finding contrasts with the results of the Hamel-Desnos study,^8^ which did not detect any significant difference in pain scores between the two groups. However, the Hamel-Desnos study had a small sample size, no formal hypothesis testing, and 40% of patients wore compression stockings. The findings are consistent with a recent systematic review that suggests the use of post-procedural compression reduces pain scores. Nevertheless, the review included patients treated with different methods such as open surgery, TTs, and foam sclerotherapy.^28^ It is essential to emphasize that, even though the statistical analysis has detected a significant difference in pain scores between the groups, the clinical significance of this difference may be limited with the use of compression stocking.

The study shows that both groups exhibited improvement in their clinical severity scores, and the degree of improvement was comparable to that reported in other studies.^28^ Consistent with previous research,^28^ the results indicate that QoL scores after foam sclerotherapy improved similarly between patients who wore compression stockings and those who did not. However, the generic QoL was significantly better in the NCG at 6 months. This finding may be attributed to the larger amount of sclerosant foam injected into the NCG, which may have contributed to a greater degree of improvement. Nonetheless, the study was not powered to detect this difference, and thus, the results may not be reliable.

The results also demonstrate that CG patients wore compression stockings for a median duration of 8 days, with a compliance rate of 45%. This does bring into question the benefit of stockings; however, the study was conducted on an intention-to-treat basis. The percentage of ecchymosis did not differ significantly between the two groups, and there was no difference between groups in the time taken to return to normal activities or work. At the 6-month follow-up, complete occlusion rates for both groups were comparable, although the study design was not powered to detect this. The incidence of complications was low in both groups, with one case each of DVT and SVT reported in the NCG, and two cases of SVT reported in the CG.

The extensive QoL data generated by this study is being used for the purpose of conducting a cost-effectiveness analysis.

### Strengths and limitations

Although the CONFETTI study was a well-designed prospective RCT with clearly defined outcome measures, several limitations must be acknowledged. Firstly, recruitment rates were lower than anticipated, with patients’ preference for TTs and the COVID-19 pandemic cited as contributing factors. As a result, the study was conducted with a smaller sample size after a revised power calculation was performed. Secondly, the high dropout rate after the 2-week follow-up period has reduced the study’s statistical power. Additionally, the impossibility of blinding the patients to the intervention was a limitation, but treatment allocation-blind follow-up assessments helped address this concern. Patient compliance with compression stockings was also suboptimal, with less than 50% adherence observed. Analgesia consumption was not reported, despite patients being advised to use over-the-counter analgesics if necessary. The majority of treated veins were tributaries, potentially limiting the study’s generalizability. Finally, the study did not assess the long-term effects of foam sclerotherapy, and future research is required to determine recurrence rates and the need for repeat procedures following the initial use of compression.

## Conclusions

The CONFETTI study findings provide evidence that the use of compression stockings following foam sclerotherapy reduces post-procedure pain in the short-term, but no significant differences were observed in the secondary outcomes. However, recruitment difficulties, compliance issues with compression stockings, and the fact that most of the veins treated were tributaries must all be considered when interpreting the results.

The study provides support for the use of compression stockings for short-term pain relief. Consequently, the current NICE guidelines for wearing compression stockings for at least 7 days post-treatment appear to be a reasonable recommendation. Future research should focus on the cost-effectiveness of post-procedural compression following UGFS as well as its long-term efficacy in terms of recurrence rates and the need for repeat procedures.

## Author Contributions

Conception and design: RB, TL, AD

Analysis and interpretation: AB, RB, TL, AD

Data collection: AB, RB

Writing the article: AB, RB, TL, AD

Critical revision of the article: AB, RB, TL, AD

Final approval of the article: AB, RB, TL, AD

Statistical analysis: AB, RB, TL, AD

Obtained funding: RB, TL, AD

Overall responsibility: AD

## Disclosures

None.

## Figures and Tables

**Supplementary Table (online only):** Exclusion criteria

• Current DVT • Varicose veins in both legs • Arterial disease (ankle-brachial pressure index less than 0.8) • Known allergy to sclerosant • Presence of C6 disease/active ulceration • Unable to wear compression stockings • Unwilling to participate • Inability to complete questionnaires or inability to attend follow-up appointments

*DVT*, Deep vein thrombosis.

**Supplementary Fig (online only):**
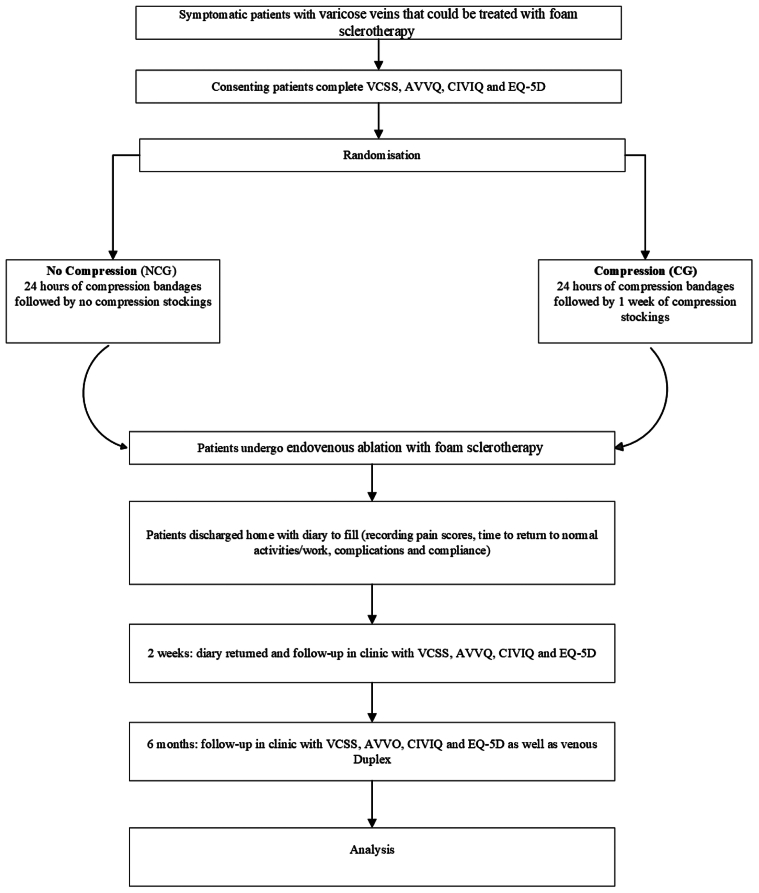
Study flow diagram. *AVVQ*, Aberdeen Varicose Vein Questionnaire; *CIVIQ-14*, Chronic Venous Insufficiency Quality of Life Questionnaire; *CG*, compression group; *EQ-5D*, EuroQoL 5 Domain 3 Level; *NCG*, no compression group; *VCSS*, Venous Clinical Severity Score.

## Appendix

CONSORT_checklist
